# Inflammatory Skin Change From a Small 0.9 cm Primary Breast Cancer Not Seen on Initial Imaging: A Case Report

**DOI:** 10.7759/cureus.39491

**Published:** 2023-05-25

**Authors:** Dallin Judd, Brenton Stucki, Jake Oldham, David Johnston

**Affiliations:** 1 Texas College of Osteopathic Medicine, University of North Texas Health Science Center, Fort Worth, USA; 2 Radiology Partners, Rose Imaging Specialists, Fort Worth, USA

**Keywords:** small breast mass, inflammatory skin changes, invasive ductal cell carcinoma, breast imaging, inflammatory breast cancer

## Abstract

Most inflammatory breast cancers are caused by invasive ductal cell carcinomas that arise from mammary epithelial cells lining the breast ducts. Typically, in these cancers, radiological signs are conspicuous, and a diagnosis is made after standard mammographic imaging or ultrasound. We report the case of a 54-year-old female who presented to a mammography clinic with right-sided breast pain and swelling. Upon physical examination, there was no palpable mass. Ultrasound and mammogram findings included mild skin thickening, normal-sized but irregularly shaped axillary lymph nodes, and no breast mass. Due to the presence of inflammatory changes (skin thickening) and abnormal lymph nodes but no obvious mass, an MRI was done to find the primary mass after a core needle biopsy of one lymph node showed metastatic ductal disease. In this patient, a 0.9 cm mass was found at the right 8:00 position on MRI. A second-look ultrasound was then performed and the mass was identified, followed by an ultrasound-guided core biopsy. The biopsy showed an invasive ductal cell carcinoma. In most cases, inflammatory breast cancer is associated with larger tumor sizes. However, a subset of patients with inflammatory breast cancer may present with a small primary breast tumor that causes inflammatory changes. Here, we present a rare case of inflammatory breast cancer associated with a small breast mass measuring less than 1 cm in size.

## Introduction

In this case report, we present an unusual case of inflammatory breast cancer where the primary mass was not visualized on the initial mammogram or ultrasound. As inflammatory breast cancer often has extensive skin changes and a large breast mass or masses, a mammogram and ultrasound are usually sufficient for primary identification. However, this case highlights the appropriate steps to take when the initial imaging is unsuccessful in locating the primary breast lesion. Our aim with this case is to highlight an atypical example of an inflammatory breast cancer presentation with the goal of helping physicians understand the additional steps available to make an accurate and timely diagnosis.

Inflammatory breast cancer is a rare and aggressive form of breast cancer, accounting for between 1% and 5% of breast cancers in the United States [1]. Clinical manifestations leading to suspicion of breast cancer typically include localized breast masses, pain, swelling, discharge, xerosis, and lymphadenopathy. Conventionally, mammography is the standard imaging modality to discover malignant breast masses. In inflammatory breast cancer, visualization of the primary breast tumor facilitates the diagnosis as the tumor can be biopsied to determine the pathology. Axillary lymph nodes, skin thickening, architectural changes, and any calcifications are also evaluated if they are present on the initial mammogram.

In the presence of abnormal axillary lymph nodes on ultrasound without a visible breast mass, further evaluation is done by ultrasound-guided biopsy of one of the nodes. If the lymph node biopsy confirms metastatic breast disease, an MRI is subsequently ordered to discover the primary lesion. A study from The University of Texas MD Anderson Cancer Center illustrated that for invasive breast cancer, MRI has the highest sensitivity in detecting primary mammary parenchymal lesions. Results of the study showed that in the presence of invasive breast cancer, MRI identifies all breast parenchymal lesions, mammography identifies 80%, and ultrasound identifies 95% [2].

MRI is utilized in the screening of high-risk patients to evaluate the extent of malignant disease or as a problem-solving tool. When mammography and ultrasound do not identify the primary breast mass and when malignancy is clinically suspected, a breast MRI is performed. If a suspicious mass is seen on MRI, a second-look ultrasound uses the localizing information from the MRI to focus the evaluation and localize the mass. It has been shown to be a useful diagnostic tool for lesions discovered with MRI [3]. If a suspicious mass seen on MRI cannot be localized with ultrasound, it can be biopsied under MRI guidance.

Historically, inflammatory breast cancer has shown a poor prognosis, with a survival rate of less than 5% beyond five years when treated with surgery or radiation therapy [4]. Inflammatory breast cancer has an incidence of around 5,000 new cases annually [4]. Due to its rarity, inflammatory breast cancer is often misdiagnosed as mastitis or generalized dermatitis, making accurate diagnosis challenging. This particular case highlights a unique clinical presentation of inflammatory breast cancer. In a five-year retrospective analysis of inflammatory breast cancer presentations on MRI, the median size of the mass lesion detected was 6 cm [5]. Here, we present a case of inflammatory breast cancer caused by an underlying mass measuring 0.9 cm.

## Case presentation

**Figure 1:**
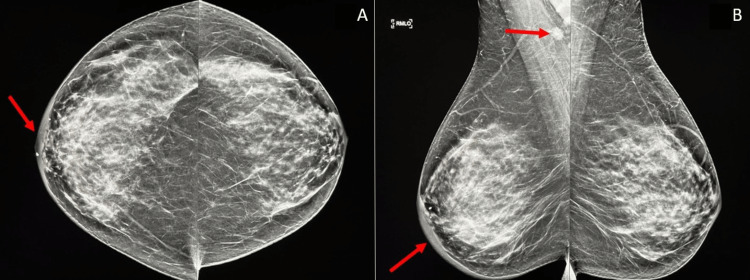
Diagnostic mammogram. A. Bilateral craniocaudal view. B. Bilateral mediolateral oblique view. Remarkable findings include skin thickening, increased anterior mammographic density (A and B), and small irregular right axillary lymph nodes (B).

A 54-year-old female presented to the mammography clinic with right-sided breast pain and swelling. The patient underwent diagnostic bilateral mammography along with targeted right breast and axillary ultrasonography. The mammogram revealed dense breast tissue, no obvious breast mass, skin thickening, and normal-sized but irregularly shaped lymph nodes in the right axilla (Figure 1).

**Figure 2:**
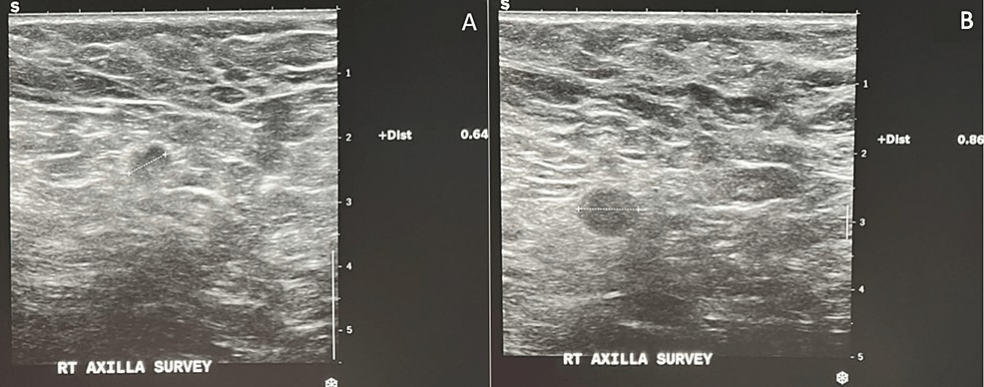
Ultrasound of the right axilla. A. A 0.64 cm lymph node. B. A 0.86 cm lymph node. Both nodes have an irregular shape and loss of their fatty hilum.

Ultrasound revealed two small (less than 1 cm) irregularly shaped lymph nodes in the right axilla along with cortical asymmetry and thickening (Figure 2). No right breast mass was discovered. One of the lymph nodes was biopsied and was positive for breast adenocarcinoma.

**Figure 3:**
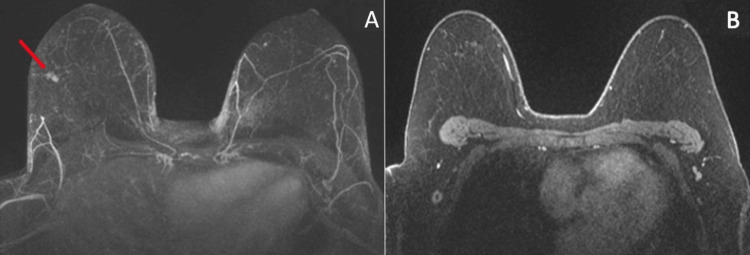
MRI findings. A. Maximum intensity projection image. B. Axial contrast-enhanced T1-weighted image. A 1 cm mass is seen at the right 8:00 position (A, red arrow). The biopsied lymph node is seen in the right axilla (B).

Due to the positive biopsy of the lymph node but no obvious primary breast lesion seen, the physician ordered a breast MRI. Breast MRI showed a 0.9 cm enhancing mass (washout type III enhancement curve) at the right 8:00 position (Figure 3).

**Figure 4:**
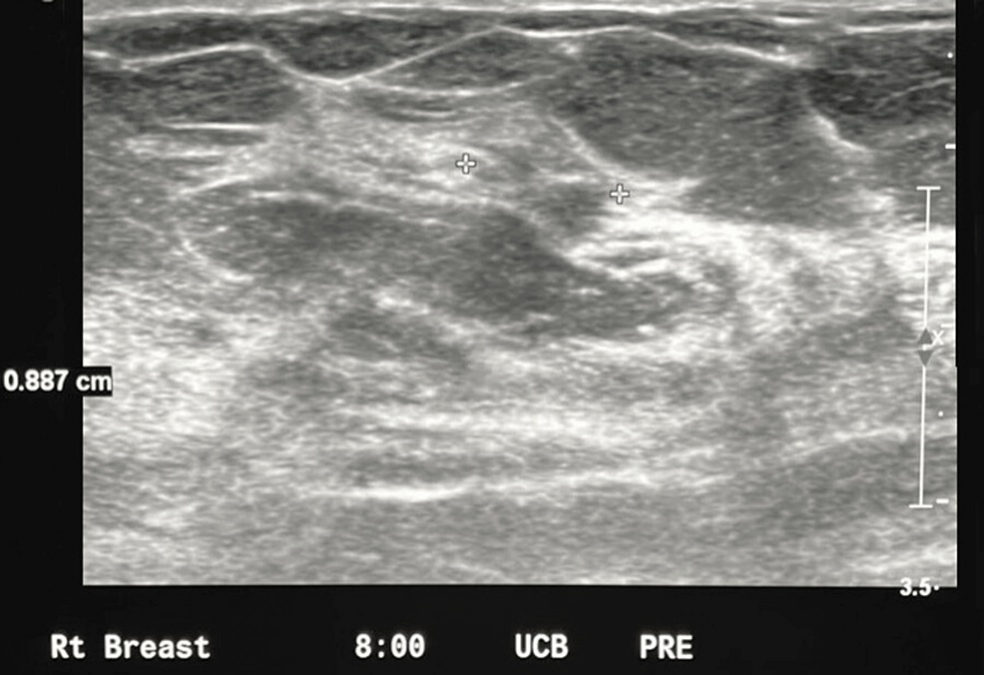
Ultrasound of the right breast. The 0.9 cm mass in the 8:00 position.

With positive findings of a mass seen on MRI, a second-look ultrasound of the right breast was performed. Second-look ultrasounds are performed after MRI as some ultrasound findings are very difficult to visualize on an initial ultrasound examination unless it is known exactly where to look. Upon performing the second-look ultrasound, a 0.9 cm breast mass was identified at the right 8:00 position and then biopsied to determine the histology of the breast finding (Figure 4). This mass was small and mostly isoechoic, making it difficult to visualize without the directed localization provided by MRI.

The biopsy of this mass returned as invasive ductal cell carcinoma (IDCC).

## Discussion

Ductal carcinoma in situ (DCIS) of the breast is quite common, accounting for about 25% of diagnosed breast cancers [6]. If caught early enough, typical presentations of DCIS have a five-year survival rate of nearly 100% [7]. In a recent 2020 study, it was concluded that 36% to 100% of DCIS cases left untreated progressed to IDCC [8]. The cancer cells spread outside the milk ducts to other parts of breast tissue and can metastasize [9]. Most inflammatory breast cancers are a form of IDCC, which usually involves the skin and dermal lymphatics [1]. Typically, inflammatory breast cancer is fairly extensive and is usually seen when mammography and ultrasound are used as combination diagnostic tools [10]. This is a case where that was not true, and additional imaging in the form of MRI was required to identify the primary mass.

IDCC most commonly presents as a non-palpable mass, making regular screening important for all women. Conversely, these carcinomas, when inflammatory, usually present with a palpable mass and skin thickening. They are typically found on imaging through hallmark features such as extensive visible cancer, background parenchymal increased density, and skin thickening. Often, lymphadenopathy is present.

In the case of this patient, the primary mass was not seen on the mammogram due to its small size. Although inflammatory markers such as skin thickening and lymphadenopathy were visualized, other typical indicators, such as a visible primary breast mass, were not seen with the initial mammogram and ultrasound. This uncommon presentation highlights the necessity for additional imaging in the form of an MRI, which aided in localizing the primary breast mass. The adherence to the initial mammography and ultrasound protocol, followed by MRI when necessary, facilitated detection and diagnosis.

Staging is a critical step in the diagnostic process for inflammatory breast cancer as it helps guide treatment decisions and predict the patient’s prognosis. The TNM staging system considers various factors, such as the size of the tumor, whether it has spread to nearby lymph nodes, and whether it has metastasized to other parts of the body. The cancer stage determines the appropriate treatment plan, including chemotherapy, surgery, radiation therapy, or a combination of these treatments. The earlier the cancer is diagnosed and staged, the better the chances of successful treatment and improved prognosis. Compared to non-inflammatory breast cancer, inflammatory breast cancer is often diagnosed later due to its unique symptoms, which can be mistaken for other conditions. Additionally, inflammatory breast cancer is a particularly aggressive type of breast cancer that grows and spreads rapidly, making early detection and prompt treatment critical to the patient’s prognosis.

Although breast MRI as a diagnostic tool was vital to this case and is available for use as a supplement to mammograms, screening MRI is usually reserved for those considered at high risk of developing breast cancer. This is because breast MRI results may appear abnormal when in reality, there is no cause for concern (a false-positive) [11]. It should be noted that the sensitivity of breast MRI has been reported to be very high (over 90%), but its specificity has been reported to be low to moderate (72%) [12]. This sometimes makes the discrimination capacity of breast MRI between benign and malignant lesions challenging. False-positive findings on breast MRI may lead to unnecessary biopsies [12]. Several screening MRI studies have reported a false-positive rate ranging from 52 per 1,000 cases to 97 per 1,000 cases, which further solidified this concern [13]. Breast MRI does not show calcium deposits (micro-calcifications), a mammographic sign of breast cancer, making the mammogram an essential part of screening [13].

Inflammatory ductal cell carcinoma without the characteristic findings of a primary mass seen on an initial mammogram or ultrasound is rare. For this reason, breast MRI is used to locate the primary mass when it is clinically suspected or abnormal findings are seen in the initial imaging studies (i.e., abnormal axillary lymph nodes). As detailed in this case, information regarding the location of the primary mass obtained with breast MRI was used in an ultrasound-guided core biopsy leading to the diagnosis of IDCC.

The case we presented is one instance where the diagnostic process required additional steps due to the lack of imaging findings. In breast cancer screening, mammograms and ultrasounds alone may not be sufficient for high-risk patients. This case highlights the role of breast MRI in the localization of a small primary breast mass.

## Conclusions

It is important to acknowledge that the presentation of inflammatory breast cancer with small breast masses can pose a diagnostic challenge, as the inflammatory changes can mimic other conditions such as mastitis or systemic edema. As a result, the diagnosis of inflammatory breast cancer may be delayed, leading to a more advanced stage of disease at the time of diagnosis. The significance of our case report lies in the rarity of inflammatory breast cancer with a small breast mass measuring less than 1 cm in size. This case underscores the importance of considering inflammatory breast cancer in the differential diagnosis of patients with skin thickening of the breast, even if no breast mass is seen with initial imaging.
